# Everybody's Page

**Published:** 1890-08-02

**Authors:** 


### Everybody's Page.

Pyoctanin has been described by Dr. Adolph Kessler, of New York, to be the "ideal antiseptic andpus destroyer;" but Dr. Kolliker, of Strasburg, says that the results he has obtained from its use are little or nothing.

A cabman has lately been deprived of his sight by "tip-cat"—that awful amusement of small boys. We do wish the authorities and the "arms of the law" would forbid these pointed pieces of wood being sent flying at random through the streets. Accidents are only one day wonders, unfortunately. People say, "How dreadful!" and that is about all they do to prevent their recurrence.

Every one will welcome success for the memorial, which has received numerous signatures from both sides of the House of Commons, to the Chancellor of the Exchequer, in favour of plainly stamping the value of every coin on its face. The similarity between the crown and the four-shilling piece is a cause of frequent vexation, and the trouble it must give to a foreigner can better be imagined than described.

A fatal accident is reported by the *N.Y. Medical Record*, resulting from an apparatus for hot-air inhalations used by a lady for the relief of pulmonary trouble. The poor woman noticed a peculiar dryness in her throat, but all the same, persevered in her inhalations. She died the next day. The thermometer in the apparatus had broken, and the mercury volatilised by the heat had poisoned her.

The twelfth annual meeting of the Church of England Burial Reform Association was held this past week. The Prince of Wales wrote to say that he hopes the "present system of burying the dead may be exchanged for one more in accordance with the requirements of the age." Population increases, open spaces decrease; we see the comparatively new cemeteries gradually hemmed in by houses and humanity, and we watch the progress cremation is gradually and steadily making, and wonder if the solution of the great difficulty will really soon be reached.

Dr. Fowler, of New York, gives a new method of artificial feeding for infants, which has, he says, met with great success. Four tablespoonfuls of rice are put into three pints of water and boiled half-an-hour, then allowed to simmer at the back of the range for the day, water being gradually added to maintain the original three pints. At night this must be strained through a colander, and placed on ice, and when cold it will form a paste. Put three tablespoonfuls of the paste to each nursing half-pint bottle of milk. The fresh rice supply must be made daily. Dr. Fowler considers that the hydrated starch granules prevent the formation of solid clots of casein, and that this preparation will be easily digested. In case of constipation, Farina should be substituted for the rice, owing to its laxative qualities.

Workers in a strong electric light are subject to a stroke—electric stroke it might be called. A person may be standing twelve or thirteen yards away from the electric arc—at which distance the thermometer will not be raised in the slightest degree—when he suddenly feels a smarting sensation on his neck, face, and forehead, his skin becomes bronzed and red, and his eyes are intensely painful with the feeling that innumerable bits of dust are under the lids, and the tears flow uncontrollably for four-and-twenty hours. On first encountering daylight the person struck is quite sightless, and even after some time everything appears quite yellow. The skin in a few days peels off in large pieces. The workers in electric light try to protect their eyes by dark glasses, but in spite of precautions these strokes are by no means rare.

It is quite evident that the Italians have suffered enough at the hands of quacks and quack dentists. A recent decree compels every one who practises dentistry or phlebotomy to be first possessed of a legal medical and surgical degree.

M. Lagneau is investigating the very small and decreasing population of France, and hopes soon to indicate what may be done to arrest the decrease. Wars, civil and foreign, consumption, and other diseases have carried off the "young hopefuls" of France during the last thirty or forty years, and the excess of births over deaths the last few years only being one in a thousand compared to 13·7 per thousand in England has not helped to improve matters.

Defective sight is said to be becoming a great deal too general in America; the increase of blindness is, in fact, said to have been four times as great as the increase of the population. Can this be true? Purulent ophthalmia of infancy has been very prevalent in institutions and poor-houses in that country, and no special precautions have been taken to prevent its spreading; at least, such is the report, which, if a true one, will account partly for the first half of our statement.

The report of the British Consul at Galveston upon the brilliant commercial future of Texas seems a trifle sanguine. A people who have been educated into the notion that they are ahead of the rest of the world in arts, science, and civilization, and that what they do not know is not worth knowing, do not strike one as being a people who are likely to make rapid progress even when blessed with the advantages of a fertile soil, immense undeveloped agricultural and mineral resources, and a good climate. Everyone has heard of Texan ranches, but Texan civilization is a novelty to all except Texans.

It is curious to note that the strong-minded (?) individual who thinks holidays are waste of time is often a striking example of the truth of that proverb about "All work and no play." We of London live in a good deal of bustle and want our autumn stocktaking of fresh scenes, ideas, and air every year. Even when the Watkin Tower pumps down all that fresh air we hear about, it won't pump down the ideas or the joy that a short change of surroundings gives us. It is the new mental material which one accumulates unwittingly on a holiday which helps quite as much as the air to refresh tired humanity.

The *Quarterly* has raised the question of the merits and demerits of "*penny* fiction," and, like most of us, recognises its utter badness. We have noticed, in select parts of London, a *halfpenny* fiction, which ought to be a degree lower. Publishers will be few who will publish healthy books at a penny, and, if they did, the buyers of penny fiction would decline them with thanks. For our part we think it quite as necessary to revile the literature which comes to the hands of young servant girls, in which dukes and ghosts and pistols and love passages are all in delightful medley—virtue getting apparently the worst of it, and vice enjoying life immensely. No drastic legislation will improve matters. Then, again, some well-meaning people think goodness lies in canting morality, and the mawkish goody-goodiness of some children's books is every bit as reprehensible as "penny dreadfuls." Reform in literature is as with most other things—each in his own sphere could do so much to elevate the literature of those around him, if he would try. A helping hand and the suggestion of some interesting book would keep many away from the demoralising influence of the only literature they have ever hitherto perused.

